# Trait Association for Flowering Time in Lentil from Global Multi-Environment Data Using GWAS and Machine Learning

**DOI:** 10.3390/plants15050779

**Published:** 2026-03-03

**Authors:** Shriprabha R. Upadhyaya, Hawlader A. Al-Mamun, Monica F. Danilevicz, Shameela Mohamedikbal, Mohammed Bennamoun, Jacqueline Batley, Kirstin E. Bett, David Edwards

**Affiliations:** 1 Centre for Applied Bioinformatics, The University of Western Australia, Perth, WA 6009, Australia; shriprabha.upadhyaya@research.uwa.edu.au (S.R.U.); hawlader.almamun@uwa.edu.au (H.A.A.-M.);; 2 School of Biological Science, The University of Western Australia, Perth, WA 6009, Australia; jacqueline.batley@uwa.edu.au; 3 InterGrain Pty Ltd., Perth, WA 6163, Australia; 4 Australian Herbicide Resistance Initiative, School of Agriculture and Environment, The University of Western Australia, Perth, WA 6009, Australia; monica.danilevicz@uwa.edu.au; 5 School of Physics, Mathematics and Computing, The University of Western Australia, Perth, WA 6009, Australia; mohammed.bennamoun@uwa.edu.au; 6 Department of Plant Sciences, University of Saskatchewan, Saskatoon, SK S7N 5A8, Canada; k.bett@usask.ca

**Keywords:** single nucleotide polymorphisms (SNPs), epistasis, SHAP, XAI

## Abstract

Flowering time is an important developmental stage in plants, influenced by multiple genes and environmental factors. Understanding its genetic basis and interaction with the environment facilitates the development of improved varieties adapted to different environments. Conventional Genome-Wide Association Studies (GWAS) have been widely used to associate genetic markers with heritable traits, but they do not inherently capture interactions among single nucleotide polymorphisms (SNPs) or between SNPs and the environment. Machine Learning (ML) approaches can model these interactions and improve trait prediction even in the presence of noise and missing data. In this study, multi-environment lentil (*Lens culinaris* Medik.) data were analysed using GWAS and two widely used ML models, Random Forest and XGBoost, to identify genetic markers associated with flowering time. Model interpretability was enhanced using Explainable AI (XAI) techniques, including SHapley Additive exPlanations. GWAS identified eight significant loci across chromosomes one, two, five and seven, with the most significant SNP located at Chr2_530433205, while ML approaches identified nine markers on chromosomes one, two, three, five and seven, with the most significant SNP at Chr7_523220088. The majority of the identified markers were linked to candidate genes for flowering, while ML also identified potential epistasis. These findings highlight ML as a powerful complementary tool to GWAS for trait association.

## 1. Introduction

Lentil (*Lens culinaris* Medik.) is a globally important pulse crop with its production increasing fivefold in the past five decades to reach 6.7 million tonnes in 2022, corresponding to an estimated gross production value of USD 4.3 billion [1]. Lentils are the third most important cool-season grain legume, cultivated in a wide range of environments, including dry conditions on the semiarid Canadian prairies [2], subtropical savannah in southern Asia and cool temperate steppes [3]. Although mostly grown in Canada and India, lentil is also produced in Turkey, Nepal, Ethiopia and Australia [4]. It is important for sustainable agriculture due to its nitrogen-fixing ability, increasing productivity and decreasing yield variability when included in crop rotation [2].

Flowering is a major developmental stage in angiosperms, marking the transition from vegetative to reproductive growth. This transition can be triggered by various environmental cues that aim to synchronise flowering with favourable conditions to optimise seed production [5]. Flowering time is a complex trait that affects plant growth and development, influenced by multiple genes and environmental factors. The wide range of flowering time observed in lentils has contributed to its adaptability across environments. In the *pilosae* lentil ecotype, adapted to South Asia’s short cropping season, photoperiod insensitive dominant alleles at loci *DTF6a* and *DTF6b* confer early flowering under short photoperiods [6]. Several other flowering-related genes have been identified in lentil [7,8], including *LcFTa1* from the *FLOWERING LOCUS T* (*FT*) gene family, which shows environment-specific differential expression under varying light quality [9]. Several flowering time genes have been identified in pea (*Pisum sativum*) and soybean (*Glycine max* [L.] Merr.), including circadian-clock related MYB transcription factor genes and light signalling genes *CCA1* and *LHY*, as well as various subclasses of *FT* genes responsible for promoting flowering [10]. Quantitative trait loci (QTLs) associated with flowering time were also identified in *Arabidopsis thaliana*, including the regulatory genes *LFY* and *AGL* involved in floral meristem identity and activation of floral organ identity genes. In addition, *CRY1*, *CRY2* and *PHYA* genes were shown to be responsible for early flowering and hypocotyl elongation by interacting with various light receptors, while *TFL* was shown to inhibit flowering [11].

Understanding the genetic basis of flowering and its interaction with the environment is an important objective in plant breeding, as it facilitates crop adaptation to new environments and the generation of new varieties better adapted to specific environments. Genome-Wide Association Studies (GWAS) are widely used for identifying genetic variants associated with agriculturally important traits. Previous GWAS study in lentils performed on the same dataset as the current study used latent variable phenotypes and identified single nucleotide polymorphisms (SNPs) associated with days to flowering and flowering-related genes [12]. The study identified two QTLs in chromosome 6, both of which contained several lentil *FT* orthologs previously reported in other studies. Several other candidate genes, including *WRKY* and *Ulp1*, were also identified [12]. However, conventional GWAS methods have limitations, including sensitivity to population structure, a reliance on large sample sizes, challenges in detecting rare variants and an inability to capture interactions between SNPs (epistasis) or between SNPs and the environment [13,14,15,16]. In multi-environment trials, environmental confounding can lead to spurious associations in GWAS [16]. These limitations suggest that a traditional GWAS may be insufficient to fully understand the underlying genetic factors that affect flowering time in lentils [12]. Several studies used Best Linear Unbiased Predictions (BLUPs) derived from mixed linear models as phenotypes in GWAS to enhance the power and reliability of detecting true genetic associations in multi-environment datasets. For example, a GWAS study was conducted using BLUPs derived from multi-environment data across 70 years and various locations in common bean (*Phaseolus vulgaris*) for over 21 phenotypes [17]. This helped explore consistent genetic effects by integrating the effects of location and year, leading to the identification of three candidate genes and known genomic regions associated with target traits. However, BLUP shrinks the genetic effects towards the population mean, which may lead to a loss of information [18].

Machine Learning (ML), with its ability to uncover hidden patterns between features and targets [19], also offers a potential solution to some limitations of conventional trait association methods. ML is versatile, offering both prediction capabilities and association ability through feature importance [19,20]. Previous studies have shown its effectiveness in predicting phenotypes from genotype datasets. For example, seven different agronomic traits, including soybean flower and pod colour and seed oil content, were predicted using ML and deep learning models [20]. The loci identified by ML overlapped with significantly associated loci identified from GWAS [20]. In soybean, ML-mediated GWAS complemented conventional GWAS to successfully identify markers associated with several traits [21]. Lentil yield prediction was performed using a hybrid model combining Multivariate Adaptive Regression Spline (MARS) for feature selection with Artificial Neural Networks (ANN) and Support Vector Regression (SVR) with radial basis kernel function [22]. Several correlated traits, including pods per plant, number of secondary branches per plant and plant height, were used as input variables to predict yield. Genomic BLUP, along with ML models, were used to predict phenotypes in yeast, rice and wheat [23]. While both SVM with a Gaussian kernel and BLUP were the best methods for the wheat and rice datasets, gradient boosting machine worked best for yeast.

Here, we used a multi-environment dataset to identify markers associated with flowering time in lentil using publicly available phenotypic and genotypic data from a study by [24]. By using BLUP values in GWAS, we accounted for environmental and temporal variables. The ML models employed to identify associated SNPs and explore their interactions revealed additional markers not captured by GWAS. Integrating Explainable AI (XAI) offered an advantage over previous strategies by providing new insights into potential interaction effects and direction of environmental influence (positive or negative effect). This integrative approach demonstrates the potential of ML methods for trait association and enhances our understanding of flowering time regulation in lentil.

## 2. Materials and Methods

### 2.1. Pre-Processing of Phenotypic Dataset

Phenotypic data for days to flower (DTF), measured for each plant as the number of days till the plant has one open flower, were downloaded from GitHub (https://github.com/derekmichaelwright/AGILE_LDP_Phenology, Accessed on 12 October 2023) from a previous study conducted as part of the Application of Genomics to Innovation in the Lentil Economy (AGILE) project [12,24]. The data were downloaded for 324 accessions evaluated at 18 site-years, from years 2016 to 2018, grown across macroenvironments—Temperate (Canada and USA), South Asia (Bangladesh, India and Nepal) and Mediterranean (Italy, Morocco and Spain) (Supplementary Table S1). Each site-year contained three replicates. A Python package, pandas v2.2.0 [25], was used to clean and merge the data. Rows with missing phenotypic data were removed from further analysis. Out of 17,496 phenotypic values (from 324 genotypes across 18 site-years and 3 replicates), null values were removed, leaving 15,591 values for the final analysis.

### 2.2. Quality Control and Filtering of the Genotypic Dataset

A total of 324 accessions, genotyped using a custom lentil exome capture array as part of the AGILE project [26], were downloaded from the KnowPulse database (http://knowpulse.usask.ca/AGILE/2) (Accessed on 12 October 2023). The genotype files contained 470,397 SNPs and 90,143 indels with a quality score > 30, Minor Allele Frequency (MAF) > 0.05 and a missing frequency < 0.1 [24,27]. These were filtered using BCFtools v1.15 [28] to contain only biallelic SNPs. Linkage disequilibrium (LD) pruning was performed using PLINK v.1.90 [29] with linkage correlation coefficient (r^2^) thresholds set at 0.7, 0.5 and 0.3. An r^2^ value of 1 indicates complete linkage, while 0 indicates no linkage. Three different thresholds were tested to assess their impact on reducing the dimensionality of the dataset by reducing marker redundancy while preserving genomic information. The LD pruned file was phased and imputed using BEAGLE v.4.1 [30,31]. Following filtration and pruning, 92,942 chromosomal markers (SNPs and indels) were used for the study.

### 2.3. Genome-Wide Association Analysis for Days to Flower

A linear mixed-effects model was implemented through the gamem_met() function in the Multi-Environment Trial Analysis (metan) R package, version 1.18.0 [32]. For BLUP analysis, germplasm name (genotype entry) was set as a random effect, while the covariates, including year, site and replicate data, were set as fixed effects. The resulting BLUPs for each genotype, estimated after accounting for environmental effects, were obtained and used as phenotypes for GWAS.

The LD pruned data with an r^2^ setting of 0.5, resulting in a total of 92,942 markers, were used to perform GWAS using General Linear Model (GLM) [33], Mixed Linear Model (MLM) [34] and Fixed and random model Circulating Probability Unification (FarmCPU) [35] statistical methods implemented in the R package, r-MVP v.1.0.6 [36]. The following settings were used to run the analysis—nPC.GLM = 5, nPC.MLM = 3, nPC.FarmCPU = 3, priority = memory, vc.method = “BRENT”, maxLoop = 10, method.bin = “static”, threshold = “0.05”. The number of principal components (nPC) was determined based on the Principal Component Analysis plot (Supplementary Figure S1). The FarmCPU method breaks the model into fixed effects and random effects, making it highly efficient in capturing the population structure on its own compared to MLM or GLM. While MLM can account for population structure and kinship, GLM solely depends on the number of PCs [37]. Therefore, a higher number of PCs was chosen for GLM compared to the rest of the models. The package r-MVP uses Bonferroni adjustments calculated as the threshold divided by the number of markers. The Quantile–Quantile (Q-Q) plot, which represents the quantiles of observed *p*-values to the quantiles of expected *p*-values, was assessed, and models exhibiting the best fit in the Q-Q plot, indicating minimal statistical inflation, were chosen for further analysis. The Manhattan plot was used to visualise SNPs with a significant association with flowering time.

### 2.4. Machine Learning Models to Predict Days to Flowering

Random Forest [38] and XGBoost v1.6.2 [39] regression models were used to predict the phenotype and identify significant SNPs using feature importance. Replicate and planting site variables were merged into a single term: Rep_Site. Marker data with a 0.5 LD pruning setting, year and Rep_Site were used as the input dataset with the DTF value set as the target variable. All models were built using Python v3.7.11 packages, and Jupyter notebook v6.5.7. Pandas v2.2.0 [25] and NumPy packages v1.26.3 [40] were used to process the dataset. The marker data were converted to a 0, 1, and 2 genotype matrix with the following encoding: 0—homozygous reference alleles, 1—heterozygous genotypes and 2—homozygous alternate alleles, while the Rep_Site variable was one-hot encoded using the one-hot encoder package on scikit-learn package v1.4.0 [41]. The dataset was split into training, validation, and holdout sets at a 70:20:10 ratio using the train_test_split function to ensure a representative distribution of genotypes and environments across split. Along with a randomly selected dataset, three site-years (Sutherland-2018, Metaponto-2017 and Jessore-2017) were used as holdout sets to evaluate the model’s prediction and generalisability. Grid search cross-validation (CV) was used on training models to find the best hyperparameters, while the *k*-fold (*k* = 10) cross-validation was employed to optimise and select the best performing model to be tested on the holdout dataset. Performance was evaluated on the holdout test using the Root Mean Square Error (RMSE) (Equation (1)) and R-squared (R^2^) values (Equation (2)). The “leave-one-out” (LOO) strategy, where separate site-years were used as holdout, was performed to analyse potential overfitting and inadvertent data leakage that may occur due to random split.

Equation (1). RMSE scores measure the difference between the predicted values and actual values used to gauge the inaccuracy of a regression model. Low scores indicate high predictive accuracy, while high scores indicate a poor predictive accuracy.

(1) $$RMSE=\sqrt{\frac{\sum_{i=1}^{n}{\left(y_{i}-{\hat{y}}_{i}\right)}^{2}}{n}\;}\;\;{\hat{y}}_{i}\;\mathrm{is}\;\mathrm{the}\;\mathrm{predicted}\;\mathrm{value}\;\;y_{i}\;\mathrm{is}\;\mathrm{the}\;\mathrm{true}\;\mathrm{value}\;\;n\;\mathrm{is}\;\mathrm{the}\;\mathrm{total}\;\mathrm{number}\;\mathrm{of}\;\mathrm{samples}$$

Equation (2). The R^2^ values represent how well the model fits the data by assessing the percentage of variation in the target variable explained by the independent variables. It helps understand the proportion of variance in the target variable, with 1 being a perfect fit and negative values indicating the model does not explain any variability.

(2) $$R^{2}=1-\frac{\sum_{i=1}^{n}{\left(y_{i}-{\hat{y}}_{i}\right)}^{2}}{\sum_{i=1}^{n}{\left(y_{i}-\bar{y}\right)}^{2}}\;\;{\hat{y}}_{i}\;\mathrm{is}\;\mathrm{the}\;\mathrm{predicted}\;\mathrm{value}\;\;\;y_{i}\;\mathrm{is}\;\mathrm{the}\;\mathrm{true}\;\mathrm{value}\;\;\;\bar{y}\;\mathrm{is}\;\mathrm{the}\;\mathrm{mean}\;\mathrm{of}\;\mathrm{predicted}\;\mathrm{values}$$

### 2.5. Identification of Significant Features Using Explainable AI

The XGBoost feature importance plot was used to identify the top features contributing to the model’s prediction using the ‘plot_importance’ function with ‘gain’ chosen as the importance_type. The XGBoost feature importance gain plot highlights the most important features (variables) the model uses to make its predictions, with a higher gain value corresponding to a stronger influence on the prediction. Feature importance for Random Forest was extracted using the ‘feature_importances’ function, and the values were plotted using a custom script available on GitHub (https://github.com/shriprabhau/Flowering_time_association_in_lentils/blob/main/Prediction_model_prune_50.ipynb). Shapley Additive exPlanations (SHAP) beeswarm and dependence plots were also used as an XAI method to understand the features, their interactions and correlation with the target [42].

### 2.6. Candidate Gene Identification

The positions of significant SNPs identified from GWAS and ML and their surrounding genomic regions of 169 kbps, defined by the LD decay region for the population [12], were examined in the annotated lentil genome using JBrowse (https://knowpulse.usask.ca/jbrowse/Lens-culinaris/2) (Accessed on 21 October 2024). The closest gene present within the LD decay region was selected as the candidate gene.

## 3. Results

### 3.1. Assessing Phenotypic Variation Across Sites

**Figure 1:**
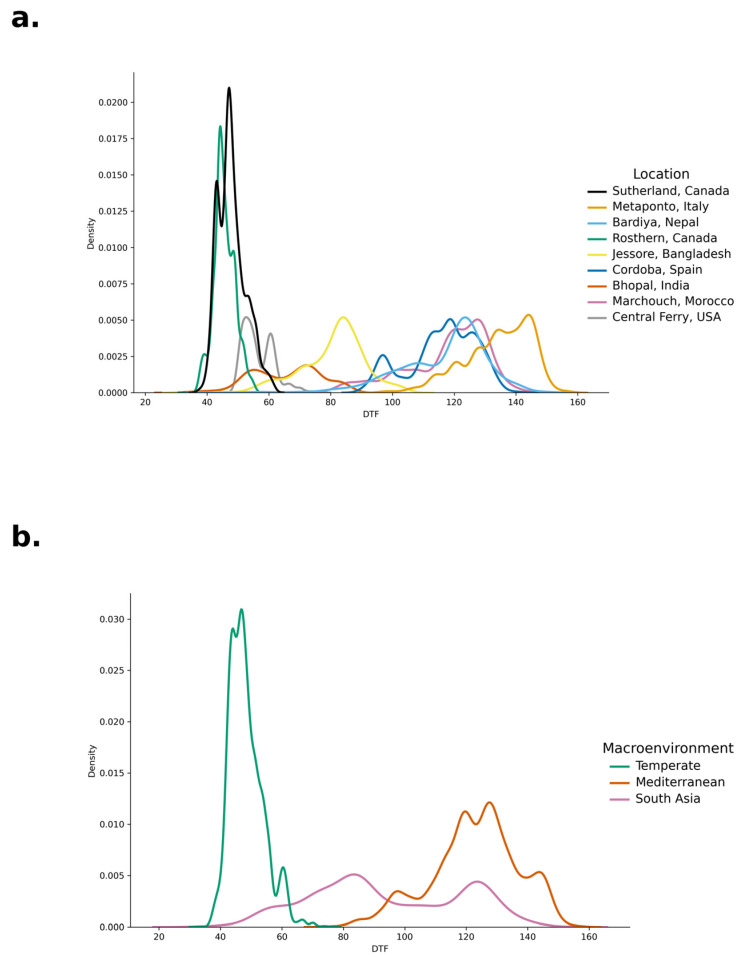
(**a**) Distribution of days to flower in lentils for each site of evaluation; (**b**) distribution of days to flower in lentils for each macroenvironment.

A wide variation was observed for DTF across all site-years (Figure 1a), with the earliest DTF observed in Rosthern, Canada, at 30 days and the latest in Metaponto, Italy, at 160 days. Several peaks were observed in the overall DTF distribution (Figure 1 and Supplementary Figure S2) [24]. Crops grown in Temperate environments showed early flowering (40–60 days), while those in Mediterranean environments showed delayed flowering (90–150 days) (Figure 1b). South Asian environments showed a broader distributed pattern from 40 to 140 days (Figure 1b).

### 3.2. Significant Markers Detected Using GWAS

**Figure 2:**
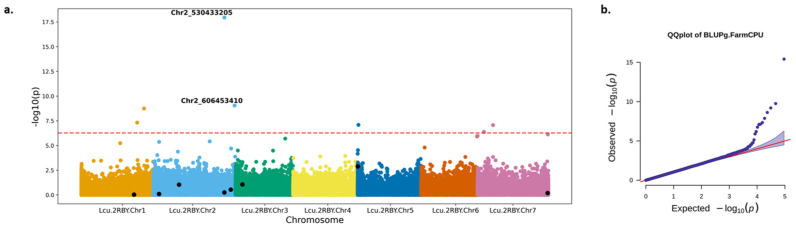
GWAS analysis using the FarmCPU method. (**a**) Manhattan plot showing significant SNPs after GWAS using FarmCPU. The dotted line indicates the significance threshold at −log10(*p*)7; the top two significant SNPs are annotated, and SNPs identified as important in ML are dotted in black. (**b**) Q-Q plot of BLUP FarmCPU GWAS results with significance at −log_10_(*p*) = 7. The red line indicates the distribution of expected *p*-values when uniformly distributed.

The Q-Q plot for FarmCPU and MLM shows that the per-SNP *p*-values closely follow the expected distribution, with deviation observed at the genome-wide threshold of significance (−log_10_(*p*) = 7), while GLM shows a deviation from the expected line before reaching the significance threshold indicating inflation (Figure 2 and Supplementary Figures S3 and S4).

GWAS using FarmCPU identified eight significant SNPs associated with flowering time (Figure 2) with a significance threshold level set at 5.38 × 10^−7^, whereas MLM identified only a single SNP that passed the Bonferroni-corrected significance threshold (Supplementary Figure S3). Therefore, the FarmCPU results are highlighted in further analysis. The most significant SNP located on chromosome 2 was identified by both FarmCPU (Chr2_530433205, *p*-value 3.85 × 10^−16^) and MLM (Chr2_530433205, *p*-value 2.63 × 10^−7^) (Supplementary Figure S3).

### 3.3. Performance of Machine Learning Models in Trait Prediction

The best ML predictions were observed using the LD pruned dataset with the use of covariates and random split, with the XGBoost model having the best R^2^ value and RMSE scores of 0.98 and 4.58, respectively, observed after cross-validation (Table 1). Using the full set of markers without additional covariates resulted in poor performance, with the random forest model performing worse due to higher dimensionality in the data (R^2^ = −0.16, RMSE = 38.53) than XGBoost (R^2^ = 0.14, RMSE = 38.16). In contrast, a reduced dataset generated after LD pruning, supplemented with covariate information, significantly enhanced the model’s performance. Using a specific site-year as a holdout showed worse performance compared to a random holdout dataset. Both XGBoost and Random Forest benefited from LD pruning; XGBoost consistently outperformed Random Forest (Table 1).

The “Predicted vs. Actual” plot for the best performing model shows tightly clustered points around the regressed diagonal line, indicating the model is performing well, while the residual plot shows no specific pattern, with mostly scattered and random points showing that the model errors are randomly distributed (Supplementary Figure S5).

### 3.4. Feature Importance and Interactions Identified Using Explainable AI

**Figure 3:**
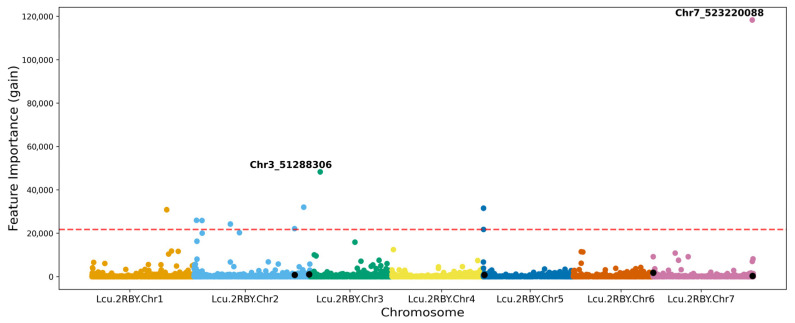
Manhattan plot illustrating feature importance scores for markers across chromosomes for the best performing XGBoost model. The top two important SNPs are annotated, and SNPs identified as significant in GWAS are dotted in black. The red dashed line represents the threshold for the top 10 features in the model.

Chr7_523220088 and Chr3_51288306 are the two most important SNPs used by the XGBoost model prediction, and ‘rep_site’ is one of the important features of the model (Figure 3 and Supplementary Figure S6), with sites in Canada and Italy appearing in the top 10 most important features. These sites come from different macroenvironments. Canada and Italy also have the shortest and longest flowering times, respectively (Figure 1). The SNPs with higher feature importance scores were present mostly on chromosome 2, consistent with the findings of [12] (Figure 3), and the genomic regions on Chromosome 2 and 7 were similar to the ones identified by GWAS (Figure 2 and Figure 3). Feature importance analysis of the Random Forest model revealed the year of planting as the topmost important feature, and most of the SNPs identified by the Random Forest model overlapped with those identified by XGBoost (Supplementary Figures S6 and S7).

**Figure 4:**
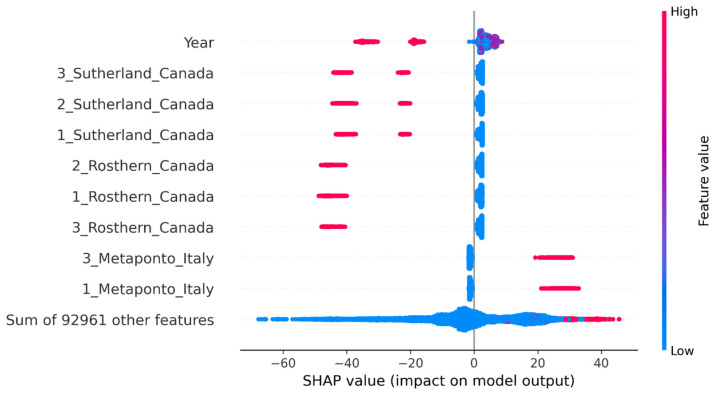
SHAP beeswarm plot showing the XGBoost model’s predictions for days to flower in lentil. The *X*-axis shows the SHAP value for each feature, representing the impact each feature has on the model’s output. The *y*-axis represents the top features. Colour gradient is the feature value for each instance, with red being higher values and blue being lower values.

SHAP beeswarm plots show that year and site are the main contributing factors for the prediction of flowering (Figure 4). The SHAP plot also shows that Canada and Italy are the top sites influencing the model’s prediction (Figure 4 and Supplementary Figure S8). It was observed that Canada negatively influenced the model’s prediction, indicating early flowering, while Italy positively affected the model, leading to late flowering. Top 25 features (Supplementary Figure S8) show that all sites from the Mediterranean environment (Italy, Morocco and Spain) affect the model positively, while Temperate and South Asian environments affect the predictions negatively, with the exception of Nepal, whose DTF values are similar to Mediterranean regions.

**Figure 5:**
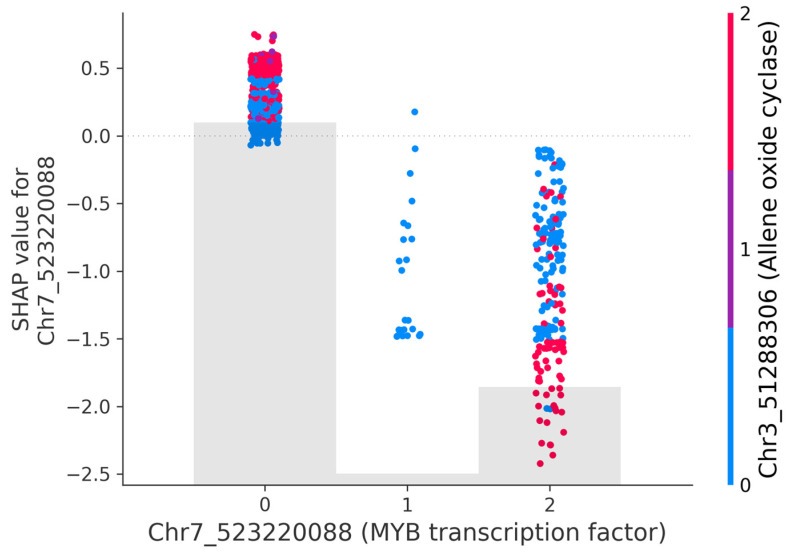
SHAP interaction plot showing the interaction for the top SNP feature. The dots represent each instance. The *X*-axis represents the feature value in the model (0—both references, 1—one alternate allele, 2—both alternate alleles). The *Y*-axis shows the effect of the feature on the model, with positive SHAP values pushing the prediction higher, while negative values lower the prediction. The colour scale indicates the value of the second feature that interacts with the top SNP feature.

The top SNP Chr7_523220088 shows a potential model-level interaction with an SNP on Chr3_51288306 (Figure 5), which is the second most important SNP picked up by the XGBoost model. Individuals homozygous or heterozygous for the alternate allele on Chr7_523220088 have negative SHAP values, while having the homozygous reference allele pushes the prediction towards positive values (Supplementary Figure S9a). However, the distribution of the positive and negative SHAP values at both 0 and 2 appears to be modulated by Chr3_51288306 (Figure 5). Both blue and red dots appear on 0 and 2; however, homozygous alternate alleles on Chr3_51288306 (red dots) are clustered at extreme SHAP values (Figure 5). This pattern indicates that the homozygous alternate allele on Chr3_51288306 may modulate the effect of Chr7_523220088, with the direction of the effect influenced by the specific allele present on Chr7_523220088. For example, when Chr3_51288306 has the homozygous alternate allele, and Chr7_523220088 has the homozygous reference allele, the model shows positive prediction, meaning the features pushed the prediction higher (delayed flowering); however, if both the SNPs have the homozygous alternate allele, the model shows negative prediction, meaning the features pushed the prediction lower (early flowering) (Figure 5). Plotting the interaction effects of other highly ranked SNPs, such as Chr1_393788469, indicate an interaction effect between the SNP and the Rep_Site feature (Supplementary Figure S9b).

### 3.5. Candidate Genes Identified Through GWAS and Machine Learning

The significant SNPs from GWAS and ML are summarised in Table 2 and Table 3, respectively. All eight significant SNPs identified using GWAS were located within or near a gene; however, the genomic regions defined by LD decay (169 kbps) lacked any annotated flowering genes (Table 2). Within the LD decay window, genes related to pigmentation and stress response were identified (Supplementary Table S2). For example, HIGH EXPRESSION OF OSMOTICALLY RESPONSIVE GENE 1 (*HOS1*)-like gene (Lcu.2RBY.Chr5:5824617..5831701), a chickpea (*Cicer arietinum*) gene homologue, lies 74 kbps from Chr5_5750212. The closest annotated flowering gene to the top GWAS SNP, Chr2_530433205, is the SUPPRESSOR OF PHYA-like gene (*LcSPA34*; Lcu.2RBY.Chr2:531140525..531145169), located 707 kbps away at the 531140525th position. Other flowering time genes, such as EMBRYONIC FLOWER 2 (*EMF2*)-like gene *(LcEMF2;* Lcu.2RBY.Chr5:5951486..5957710), *LcCDF45c* (Lcu.2RBY.Chr7:115,269,066..115,271,844) and Squamosa promoter-binding-like protein 8 gene (*LcSPL8*; Lcu.2RBY.Chr7:489,740..492,090), a chickpea homologue, were found within 500 kbps interval of the significant SNPs. Chr2_42638733 was 173 kbps away from a previously identified flowering time QTL in this population [12]. Additional flowering time genes, such as Flowering Locus K-like gene (*LcFLKa*; Lcu.2RBY.Chr6:416,287,186..416,293,464) and CONSTANS-LIKE 5 (*LcCOLd;* Lcu.2RBY.Chr7:523428965..523431132) were found within a 1.5 Mbps region of the significant SNPs.

The top SNPs for XGBoost and Random Forest models are summarised in Table 3. All identified SNPs corresponded to a gene within the LD decay window (Table 3). The closest flowering time gene to Chr7_523220088, identified by both the RF and XGBoost models, is the zinc finger protein CONSTANS-LIKE 5 (*LcCOLd*; Lcu.2RBY.Chr7:523428965..523431132), a chickpea gene homologue, positioned 208 kbps away. Chr5_1086164 lies within a previously characterised flowering time QTL, while Chr2_41685069 is 780 kbps away from another known flowering time associated region, both previously reported for this population [12]. Chr2_529882038 is situated between two flowering time genes—Squamosa promoter-binding-like protein 3 gene (*LcSPL3d*; Lcu.2RBY.Chr2:528,723,741..528,725,534) and SUPPRESSOR OF PHYA like (*LcSPA34*; Lcu.2RBY.Chr2:531,140,525..531,145,169) present 1.1 Mbps and 1.2 Mbps away, respectively. Around the LD decay window area, plant development and disease resistance genes such as Pentatricopeptide Repeat (PPR) containing plant protein (Lcu.2RBY.Chr2:578206769..578209551) present 64 kbps downstream of Chr2_ 578270690, were identified (Supplementary Table S2).

While a direct overlap of loci was not observed between GWAS and ML, some of the SNPs identified by both GWAS and ML have the same flowering time genes identified around them. For example, Chr2_530433205 from GWAS and Chr2_529882038 from ML, present 551 kbps from each other, identified the *LcSPA34* gene, while Chr7_524888573 from GWAS and Chr7_523220088 from ML, present within a 1.7 Mbps region of each other, have the flowering gene *LcCOLd* gene around them.

## 4. Discussion

This study aimed to predict days to flower in lentils using global multi-environment data. A combination of BLUP with GWAS and ML models was used to associate markers with flowering time. ML can effectively complement GWAS by broadening the scope of conventional GWAS methods, capturing non-linear effects, and effectively handling multi-environmental data. In plants, flowering at the correct time is important for the proper development of seeds and fruits, as it directly impacts crop productivity and adaptation [5]. Generally, flowering is influenced by both the plants’ genetic makeup and the environment in which it is grown; therefore, studying DTF collected from multiple environments offers an understanding of flowering time regulation and crop adaptation [12,24]. Plants interact with and react to numerous biotic and abiotic factors continuously to regulate their growth and development [43]. Here, we used the site and year of planting as covariates to account for environmental variability while determining significant SNPs.

The SNPs identified in this study by both GWAS and ML have flowering time genes or QTLs located within a 1 Mbps region, validating their significance. Various annotated stress response and resistance genes were also present within the LD decay window, many linked to flowering. For example, the *HOS1*-like gene associated with the GWAS SNP (Chr5_5750212) acts as a negative regulator of cold stress responses. Under cold stress, HOS1-mediated ubiquitination mechanism degrades CONSTANS (CO), a central activator of photoperiodic flowering, suppressing FT and delaying flowering in *A. thaliana* [44]. Similarly, PPR proteins identified by Chr2_578270690 and Chr7_115649259, primarily known for organelle biogenesis, plant development, and environmental stress responses, can indirectly influence flowering and fertility by regulating the expression of Cytoplasmic Male Sterility related genes [45,46]. Overall, the majority of the significant SNPs have stress response and resistance genes within close genomic regions, supporting prior studies that demonstrate a strong association between stress response and flowering regulation [47].

A previous GWAS study was conducted on the same dataset where multiple statistical models, including BLINK, MLM and MLMM, were used on latent variable phenotypes to understand flowering in lentils [12]. Notably, the top SNP (Chr2_530433205) identified by both FarmCPU and MLM methods and SNP Chr2_606453410 identified by FarmCPU were reported as significant in the prior analysis [12]. The repeated identification of Chr2_530433205 across various statistical models underscores its strong association with flowering time regulation in lentils. The SNP lies within a gene encoding the Multidrug and Toxic Compound Extrusion protein, which transports various substrates and contributes to diverse physiological functions, including flavonoid transport and accumulation in flowers, as seen in *A. thaliana* and *Medicago truncatula* [48,49,50,51]. Chr2_606453410 is associated with a Basic helix-loop-helix (bHLH)-like protein transcription factor that modulates its target genes by binding to specific promoter sites, influencing photomorphogenesis and flowering induction [52,53]. These factors are also responsible for petal size control [54] and pistil development in *A. thaliana* [55], indirectly affecting *FT* regulation and expression, by controlling light signalling [5,56,57]. In lentils, bHLH transcription factors, alongside *FT* genes, play an important role in regulating flowering time under varying light environments [9]. Another marker identified by GWAS, Chr7_524888573, is associated with Transducin/WD40 repeat protein. FVE WD40 repeat protein is known to play a role in flowering and floral development in *A. thaliana* [58,59].

An overlap of loci between GWAS and ML was not observed; however, there was an overlap in some of the genomic regions and corresponding flowering genes, such as the *LcCOLd* gene, reflecting the potential of ML approaches. GWAS and ML identifying markers located near the same flowering gene highlights this genomic region as a strong candidate for flowering time QTL. Similar to GWAS, ML models identified SNPs with putative links to flowering time regulation. Chr7_523220088 and Chr2_529882038 were identified by both the XGBoost and Random Forest models, further emphasising their importance. Notably, the SNP Chr7_523220088, ranked as the top feature by both models, is located within a MYB transcription factor gene and is also close to a flowering time gene. MYB transcription factors are versatile regulators in the flowering process, influencing floral organ development, colouration and control of transition to flowering via circadian-clock related genes such as *CCA1* and *LHY* in *A. thaliana*, pea, and other species [5,10,11,60,61]. In chickpea, the MYB transcription factor is encoded by the *SFL* (single flower) gene, whose mutation regulates inflorescence architecture [62]. MYB21 and MYB24 are important in regulating stamen development, with *myb21* null mutants inducing male sterility in *A. thaliana* [63,64,65]. This is evidenced by the SHAP plot showing the effect of Chr7_523220088 on flowering time, where mutation (homozygous alternate alleles) impacts flowering negatively. Another significant ML model feature, Chr3_51288306, co-located with Allene oxide cyclase (AOC), is an important enzyme involved in the jasmonic acid (JA) biosynthesis pathway [66]. JA, a plant hormone, acts as a signal regulating stress responses [67] and various developmental processes, including stamen development [68], pollen maturation [69] and flowering [70]. JA also influences flower development, with AOC1 and AOC4 showing partial promoter activity during flower development in *A. thaliana* [71].

Beyond SNPs, the top features identified by the XAI methods were the year of planting and site, with Canada and Italy influencing the model’s prediction heavily. While Canada indicated a negative influence on model prediction, Italy showed a positive influence. Although SHAP highlights the specific sites, it reflects an overall strong location effect on DTF. Canada had more observations with replicates planted across three years (2016–2018), which may have influenced the predictions; however, the number of observations alone cannot explain the effect, as Italy had fewer observations. While genotype by environment interaction (GxE) was not explicitly modelled, SHAP plots suggest that the model identified potential environmental effects. This may be due to the model’s capacity for learning hidden patterns, allowing it to identify hidden GxE signals. However, ML models cannot explicitly distinguish between the main effect and the interaction. Therefore, an explicit GxE interaction term may enhance its ability to identify environmental effects.

XAI shows a joint influence of two features on model prediction, Chr7_523220088, encoding for MYB transcription factor, and Chr3_51288306, linked to Allene oxide cyclase (AOC) involved in the JA biosynthesis pathway. This model-level interaction may indicate a potential biological interaction or epistasis between the two features. JA is known to degrade Jasmonate Zim-Domain proteins, which repress MYB transcription factors [63,72]; this interaction suggests a regulatory feedback loop influencing flowering time. Previous studies in *A. thalina* show that JA induces *MYB21* and *MYB24* activity, which controls essential aspects of anther development and filament elongation [64,69,73,74], while in tomato, JA stimulates *SlMYB21* required for coordinated flower opening and fertility [74]. In lentils, the identified model-level interaction between MYB transcription factor and AOC on flowering time provides a hypothesis for the potential regulatory crosstalk between the plant hormone and transcriptional control. This result introduces a unique hypothesis of epistasis to previous QTL identification studies. ML methods such as Random Forest have been shown to capture epistatic interactions [75,76], and our findings further support this conclusion.

Between the two ML models evaluated in this study, the XGBoost model outperformed Random Forest in flowering time prediction, achieving a lower RMSE score and thus a lower discrepancy between the predicted and observed values. XGBoost is widely recognised for its robustness and computational efficiency compared to many alternative models [20,39]. Although low R^2^ values typically indicate the model’s limited ability to explain the total variance, in this study, they were partly due to the narrow range of the dependent variable in the specific holdout (site-year) test sets, compared to the training set. In such cases, where the dependent variable has a restricted range, even small prediction errors can yield relatively low R^2^ values, despite reasonable overall model performance. While this highlights a limitation in the model’s generalisability to novel environments, the model lacked location-specific environmental covariates, limiting its ability to learn location-specific patterns. Incorporating specific environmental features may improve the model’s ability to generalise to new environments. Due to the presence of high variation in the DTF values across environments and years, a random holdout set following a similar DTF distribution trend to the training set provides a more reliable evaluation, ensuring representativeness and unbiased generalisation.

Dimensionality reduction is an important step in ML, as it improves performance by eliminating any irrelevant or redundant information [77,78] while decreasing the computational load. In this study, when using the full set of markers without LD pruning and additional covariates, both models performed poorly, suggesting that redundancy among markers from LD introduced noise, limiting the models’ ability to identify meaningful patterns. Moreover, the absence of covariates likely left out critical explanatory variables, further reducing the predictive accuracy. LD pruning reduced the complexity of the dataset by removing correlated markers, enabling the models to focus on the most informative features, while covariates captured the environmental and biological effects that were not directly encoded in the markers.

Although both ML and GWAS can detect SNPs influencing a phenotype, they differ substantially in their approach. While GWAS does detect markers with statistically significant associations to the trait, further analysis is required to understand whether the effect is additive, dominant or epistatic [13]. In contrast, ML models prioritise prediction accuracy over statistical associations between features and the target. As a result, the loci identified by the two methods did not overlap, as they are capturing different aspects of the underlying genetic architecture. Therefore, the study highlights the consistency between GWAS-detected markers and the previous literature and the potential of ML to also detect associations of markers with the trait of interest through feature importance.

A high R^2^ value of 0.98 was observed for the models, which may indicate overfitting or data leakage during random split, with the same genotype grown in a different environment being in both training and testing sets, potentially inflating the performance metrics. However, in the absence of additional environmental variables, it was difficult to fully assess the LOO strategy and conclusively determine whether the model was “remembering” the environment. It was highlighted in a previous study that used ML and GWAS to uncover candidate genes that solely relying on validation metrics for association studies can be misleading [79]. Therefore, further study by including more environmental features and a rigorous validation scheme is necessary. Although the study identified several potential SNPs linked to candidate genes, an experimental validation would be required to confirm their role in flowering. While an overlap was observed between loci identified in the current study and QTLs from a previous study [12], further functional validation in independent populations is required to confirm their effects on DTF. Therefore, GWAS and ML serve as a preliminary tool in the identification of significant markers that can be further refined through various approaches, including local haplotyping using tools such as crosshap [80]. Similar studies in soybean have used local haplotyping to understand the effects of SNPs on flowering time variation [81]. Environmental interactions, another major aspect of flowering, were not included in the study. The effect of the environment and its interaction with SNPs on flowering can be investigated further. The model’s performance could be further improved by feature selection, thereby improving the model’s association ability [22,82].

## 5. Conclusions

The study demonstrates that ML could act as a powerful complementary tool to support GWAS by identifying SNPs that are associated with flowering, especially while working with non-uniform data collected from multi-environment trials. ML complemented GWAS by capturing potential non-linear patterns, interaction effects and environmental contribution not explicitly modelled in conventional GWAS. While GWAS identified statistically significant associations, ML leveraged the dataset to uncover additional markers that may reflect the complex underlying genetic architecture, expanding our understanding of flowering time in lentils. Evidence suggests that ML can find complex hidden patterns between features in a high dimensional dataset, which is supported by our models identifying loci related to flowering. However, a common critique involving ML is its black box nature. In this study, we have used interpretable ML models along with XAI methods such as SHAP to provide further explanations. While using BLUP to account for environmental variables enables GWAS to identify SNPs that are indirectly linked to flowering, ML shows an added advantage of revealing interactions and potential epistasis between SNPs. GWAS identified several SNPs and linked candidate genes, whereas ML offered insights into the direction (positive or negative effect) of each environment’s contribution to the trait using SHAP values.

## Figures and Tables

**Table 1:** Machine learning model evaluation metrics for flowering time prediction in lentils.

Model Name	Full SNP Set Without Covariates (Random Sampling)		LD Pruned SNP Set with Covariates (Random Sampling)		LD Pruned SNP Set with Covariates (Site-Year as Holdout)	
	R^2^ Score	RMSE	R^2^ Score	RMSE	R^2^ Score *	RMSE *
Random Forest	−0.16	38.53	0.98	4.84	−1.00	7.18
XGBoost	0.14	38.16	0.98	4.58	−0.48	6.63

* Mean of three site-years as holdout. Abbreviations: LD—linkage disequilibrium; RMSE—root mean square error.

**Table 2:** Top 10 SNPs obtained from GWAS FarmCPU method.

Chromosome	Position	FarmCPU *p*-Value	SNP Location on the Genome	Gene Product Description	Gene ID
**2**	530433205	1.13 × 10^−18^	On gene	Protein DETOXIFICATION—Multidrug and toxic compound extrusion protein	ECO:0000256
**2**	606453410	9.18 × 10^−10^	On gene	Transcription factor bHLH122-like protein	ECO:0000313|EMBL:AES67711.1
**1**	468112610	1.78 × 10^−9^	On gene	Non-lysosomal glucosylceramidase	ECO:0000313|EMBL:AES60952.2
**1**	417121603	4.79 × 10^−8^	Downstream	Uncharacterised protein	ECO:0000313|EMBL:AES61566.1
**5**	5750212	8.16 × 10^−8^	On gene	DNA mismatch repair MSH4-like protein putative	ECO:0000313|EMBL:AES94295.2
**7**	115649259	8.63 × 10^−8^	On gene	PPR containing plant-like protein	ECO:0000313|EMBL:AET03769.1
**7**	48158995	4.11 × 10^−7^	Upstream	tRNA-specific adenosine deaminase	ECO:0000313|EMBL:KEH18903.1
**7**	524888573	6.51 × 10^−7^	On gene	Transducin/WD40 repeat protein	ECO:0000313|EMBL:AES92459.1
**7**	894591	1.04 × 10^−6^	On gene	Auxin efflux carrier family protein	ECO:0000313|EMBL:KEH17832.1
**6**	417490226	1.19 × 10^−6^	On gene	Carboxyl-terminal peptidase	ECO:0000313|EMBL:AES82703.1

**Table 3:** Top SNPs picked up by XGBoost and Random Forest model.

Chromosome	Position	Model	Location of the Gene	Gene Name	Gene ID
**7**	523220088	XGBoost, Random Forest	On gene	Myb transcription	ECO:0000313|EMBL:AES92359.1
**3**	51288306	XGBoost	On gene	Allene oxide cyclase	ECO:0000313|EMBL:KEH21777.1
**2**	578270690	XGBoost	Upstream	E3 ubiquitin-protein ligase RMA1H1-like protein	ECO:0000313|EMBL:KEH39118.1
**5**	1086164	XGBoost	On gene	Hypothetical protein	
**1**	393788469	XGBoost	On gene	ATPase—TCP-1/cpn60 chaperonin family protein	ECO:0000313|EMBL:ABE86688.1
**2**	12650724	XGBoost	On gene	Armadillo/beta-catenin-like repeat protein putative	ECO:0000313|EMBL:AES64018.1
**2**	41685069	XGBoost	On gene	Sec14p-like phosphatidylinositol transfer family protein	ECO:0000313|EMBL:KEH37089.1
**2**	191361888	XGBoost	On gene	Kunitz type trypsin inhibitor/Alpha-fucosidase	ECO:0000313|EMBL:AES75792.1
**2**	529882038	XGBoost, Random Forest	Downstream	Ulp1 protease family carboxy-terminal domain protein	ECO:0000313|EMBL:AES66041.1
**5**	45346120	Random Forest	Upstream	Putative ovule protein	ECO:0000313|EMBL:JAP23747.1
**1**	403726778	Random Forest	Downstream	Indole-3-acetic acid-induced protein ARG2	ECO:0000313|EMBL:KHN08677.1

## Data Availability

The original genotype data presented in the study are openly available in the KnowPulse data portal at http://knowpulse.usask.ca/AGILE/2 (Accessed on 12 October 2023), and the phenotype data are available in GitHub at https://github.com/derekmichaelwright/AGILE_LDP_Phenology (Accessed on 12 October 2023). All code generated for the study is available in GitHub at https://github.com/shriprabhau/Flowering_time_association_in_lentils (Accessed on 11 April 2025).

## Supplementary Materials

The following supporting information can be downloaded at https://www.mdpi.com/article/10.3390/plants15050779/s1, Figure S1 Principal component analysis plot; Figure S2 Graph representing data distribution for initial pre-BLUP dataset with original phenotype values on *x*-axis; Figure S3 GWAS analysis using MLM method. (a) Manhattan plot for GWAS using MLM showing significant SNPs. The dotted line indicates the significance threshold at −log10(p)7; (b) Q-Q plot of BLUP MLM GWAS results with significance at −log10(p) = 7. The red line indicates the distribution of expected *p*-values when uniformly distributed; Figure S4 GWAS analysis using GLM method. (a) Manhattan plot for GWAS using GLM showing significant SNPs. The dotted line indicates the significance threshold at −log10(p)7; (b) Q-Q plot of BLUP GLM GWAS results with significance at −log10(p) = 7. The red line indicates the distribution of expected *p*-values when uniformly distributed; Figure S5 Model evaluation plots. (a) Predicted vs. actual plot with actual phenotype values on *x*-axis and model’s predicted phenotype value on y axis. (b) Residual plot with *x*-axis showing the actual phenotype values and *y*-axis showing the residual values. The red dotted line is at y = 0; Figure S6 Feature importance gain plot for the best performing XGBoost model; Figure S7 Feature importance plot for the Random Forest model highlighting the top features of the model; Figure S8 Top 25 features influencing models’ prediction based on SHAP values. The *X*-axis shows the SHAP value for each feature, representing the impact each feature has on the model’s output. *Y*-axis represents the top features. Colour gradient is the feature value for each instance, with red being higher values and blue being lower values; Figure S9 SHAP plots for (a) influence of Chr7_523220088 on target trait and (b) interaction of Chr1_393788469 with environment. The dots represent each instance. The *X*-axis represents the feature value in the model (0—both references, 1—one alternate allele, 2—both alternate alleles). The *Y*-axis shows the effect of the feature on the model, with positive SHAP values pushing the prediction higher, while negative values lower the prediction. The colour scale for 7b. indicates the value of the interacting feature; Supplementary Table S1 Covariate data description; Supplementary Table S2 Candidate genes present within genomic intervals identified by linkage disequilibrium decay [44,45,83,84,85,86,87,88,89,90].
