# Supplementary material for: Trait Association for Flowering Time in Lentil from Global Multi-Environment Data Using GWAS and Machine Learning
Source: Plants (Basel). 2026 Mar 3;15(5):779. doi: 10.3390/plants15050779 (PMC12987266; doi:10.3390/plants15050779)
Supplement: Supplementary file 1 [file plants-15-00779-s001.zip › plants-4163705-Supplementary file.pdf]

Supplemental file for:

Trait association for flowering time in lentil from global multi-environment data using GWAS and machine learning

Shriprabha R. Upadhyaya [1,2], Hawlader A. Al-Mamun [1,2,3], Monica F. Danilevicz [4], Shameela Mohamedikbal [1,2], Mohammed Bennamoun [5], Jacqueline Batley [2], Kirstin E. Bett [6] and David Edwards [1,2] *

[1] Centre for Applied Bioinformatics, The University of Western Australia, Perth, WA 6009
[2] School of Biological Science, The University of Western Australia, Perth, WA 6009
[3] InterGrain Pty Ltd, Perth, Western Australia, 6163, Australia
[4] Australian Herbicide Resistance Initiative, School of Agriculture and Environment, The University of Western Australia, Perth, WA 6009
[5] School of Physics, Mathematics and Computing, University of Western Australia, Perth, Western Australia, Australia
[6] Department of Plant Sciences, University of Saskatchewan, Saskatoon, SK, S7N 5A8 Canada

*Corresponding author: dave.edwards@uwa.edu.au

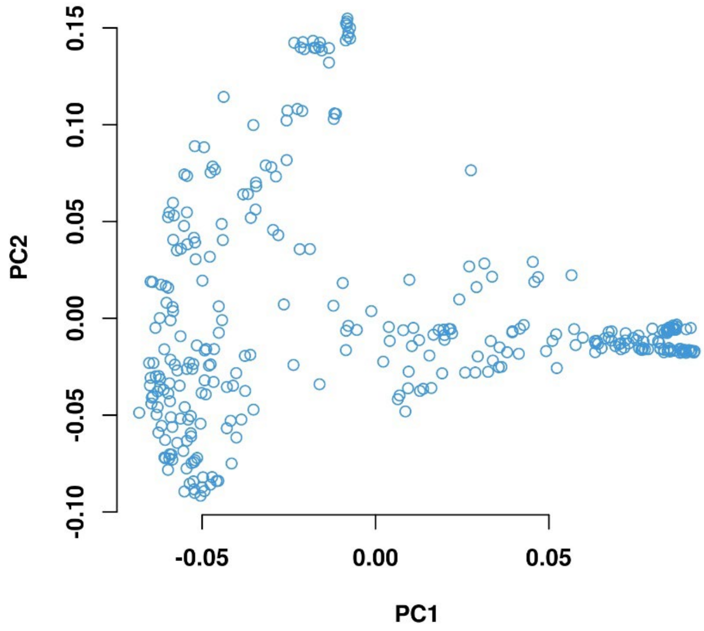

Figure S1. Principal component Analysis plot

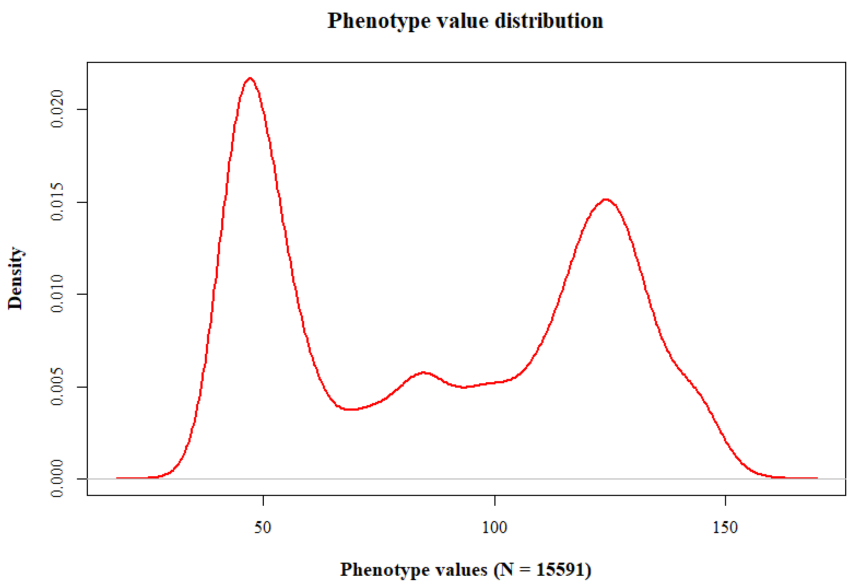

Figure S2. Graph representing data distribution for initial pre-BLUP dataset with original phenotype values on x-axis

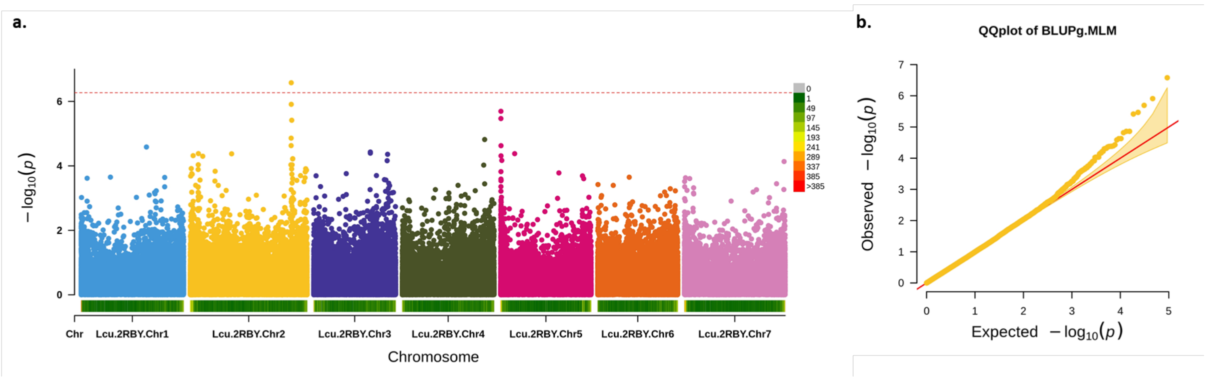

Figure S3. GWAS analysis using MLM method. (a) Manhattan plot for GWAS using MLM showing significant SNPs. The dotted line indicates the significance threshold at $-\log_{10}(p)7$; (b) QQ plot of BLUP MLM GWAS results with significance at $-\log_{10}(p) = 7$. The red line indicates the distribution of expected p-values when uniformly distributed

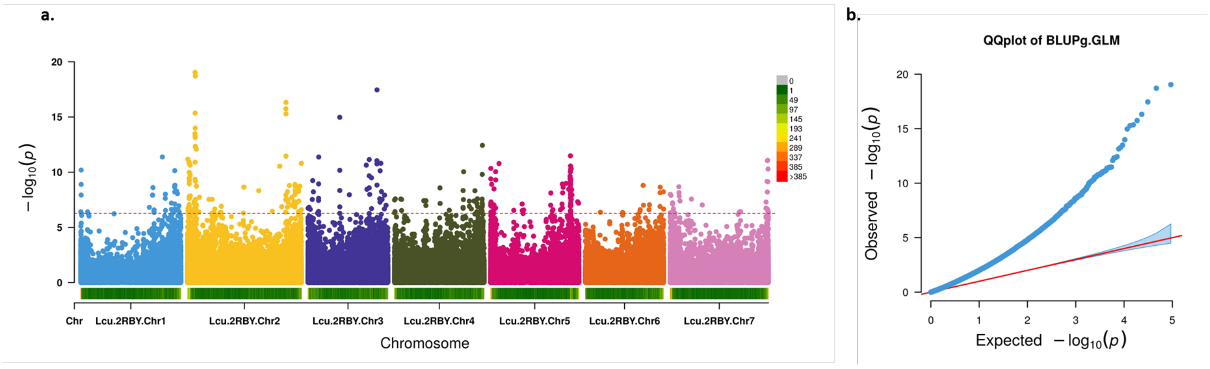

Figure S4. GWAS analysis using GLM method. (a) Manhattan plot for GWAS using GLM showing significant SNPs. The dotted line indicates the significance threshold at $-\log_{10}(p)7$; (b) QQ plot of BLUP GLM GWAS results with significance at $-\log_{10}(p) = 7$. The red line indicates the distribution of expected p-values when uniformly distributed

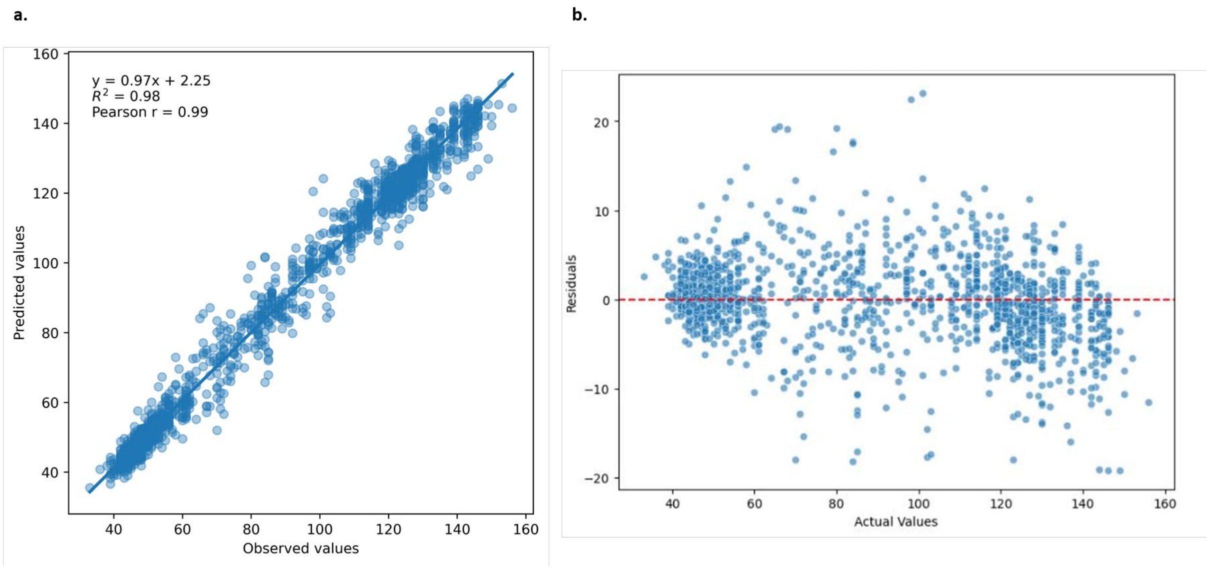

Figure S5. Model evaluation plots. (a) Predicted vs Observed plot with actual phenotype values on x-axis and model's predicted phenotype value on y axis; (b) Residual plot with x-axis showing the actual phenotype values and y-axis showing the residual values. The red dotted line is at y=0

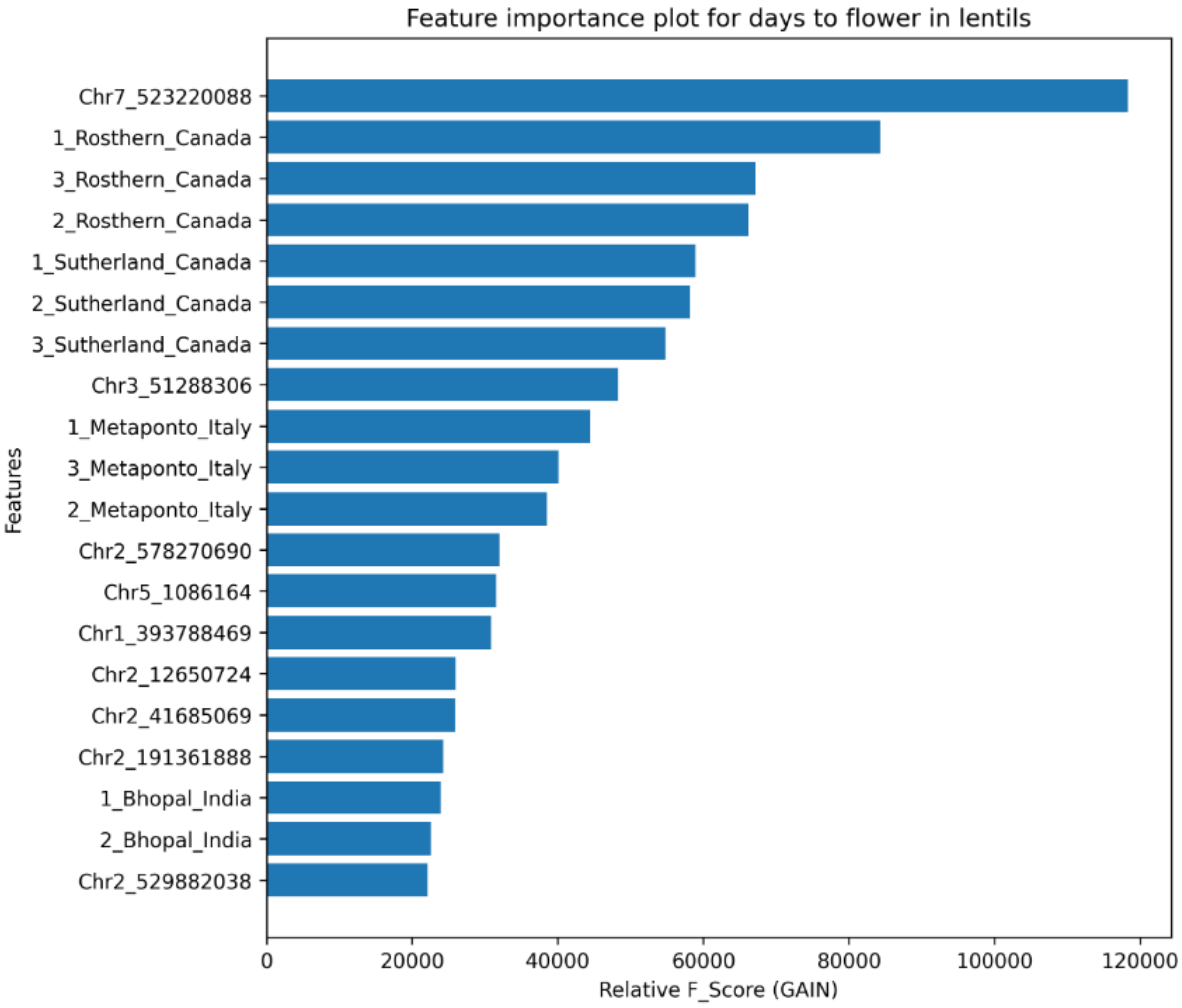

Figure S6. Feature importance gain plot for the best performing XGBoost model

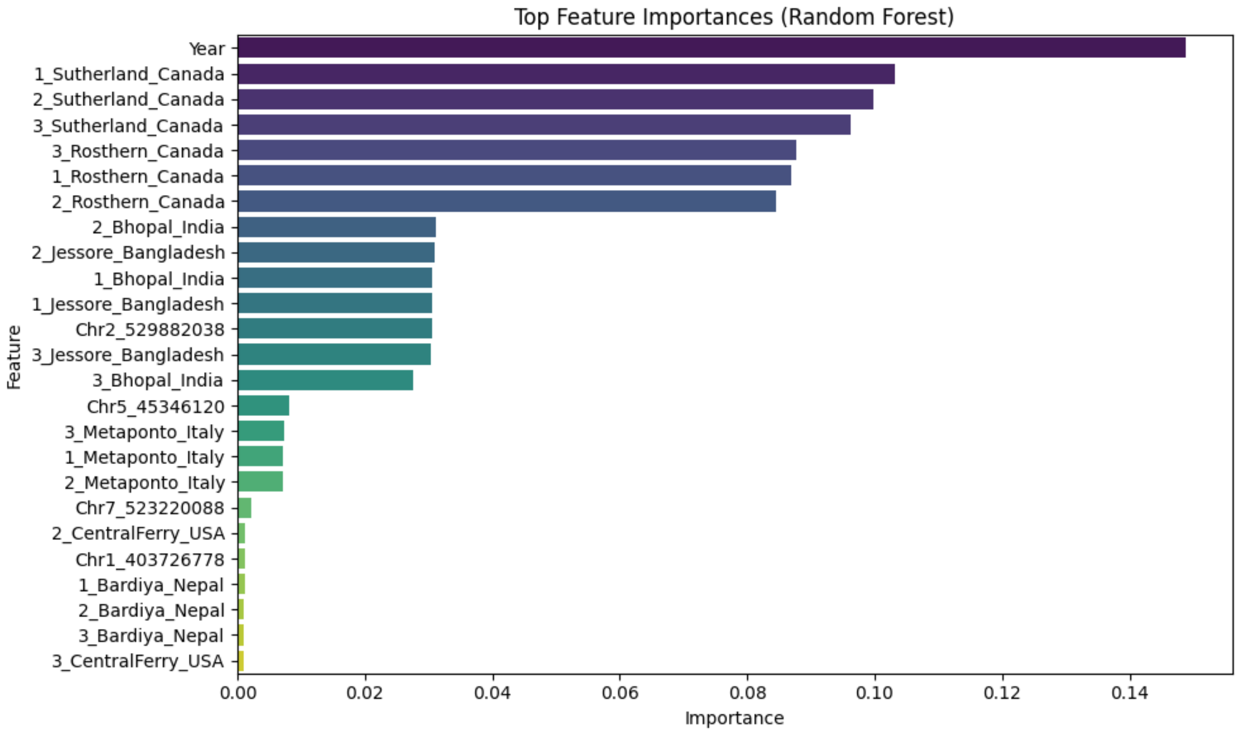

Figure S7. Feature importance plot for the Random Forest model highlighting the top features of the model.

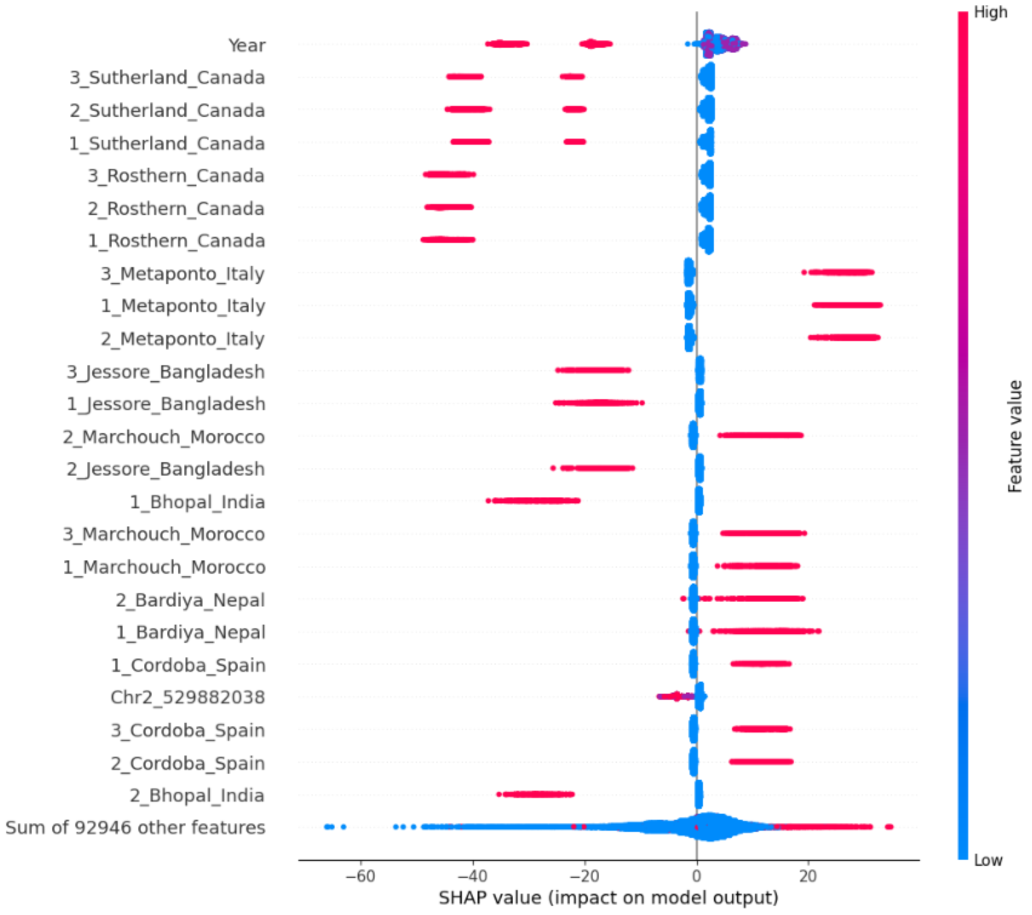

Figure S8. Top 25 features influencing models' prediction based on SHAP values. The X-axis shows the SHAP value for each feature, representing the impact each feature has on the model's output. Y-axis represents the top features. Colour gradient is the feature value for each instance with red being higher values and blue being lower values

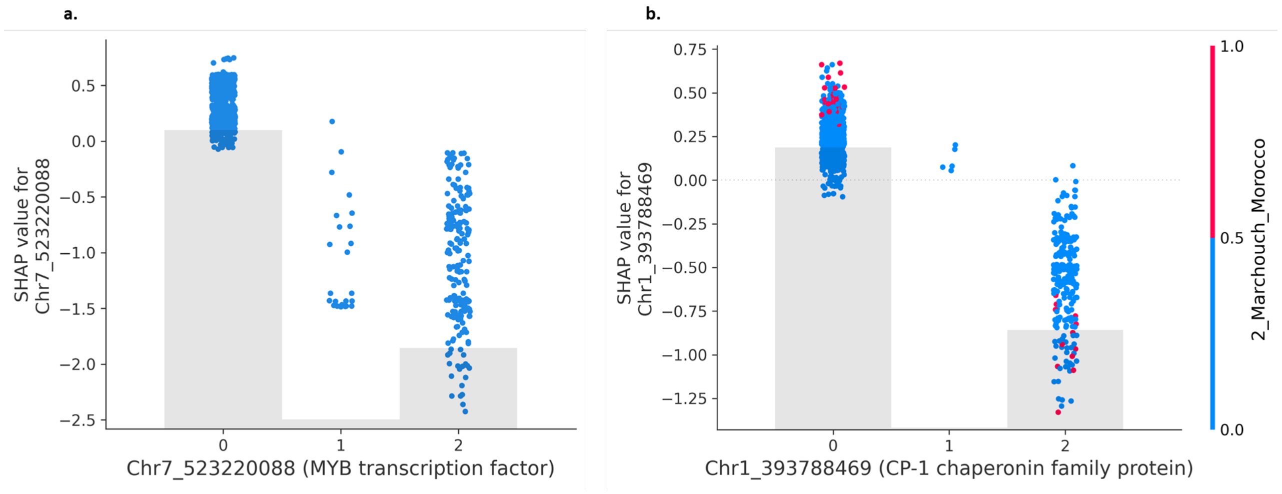

Figure S9. SHAP plots for (a) Influence of Chr7_523220088 on target trait; (b) Interaction of Chr1_393788469 with environment. The dots represent each instance. The X-axis represents the feature value in the model (0 – both references, 1 – one alternate allele, 2 – both alternate alleles). The Y-axis shows the effect of the feature on the

model, with positive SHAP values pushing the prediction higher, while negative values lowering the prediction. The colour scale for 7b. indicates the value of the interacting feature
